# Electrospun Flexible Coaxial Nanoribbons Endowed With Tuned and Simultaneous Fluorescent Color-Electricity-Magnetism Trifunctionality

**DOI:** 10.1038/srep14052

**Published:** 2015-09-16

**Authors:** Hong Shao, Qianli Ma, Xiangting Dong, Wensheng Yu, Ming Yang, Ying Yang, Jinxian Wang, Guixia Liu

**Affiliations:** 1 Key Laboratory of Applied Chemistry and Nanotechnology at Universities of Jilin Province, Changchun University of Science and Technology, Changchun 130022, China

## Abstract

In order to develop new-typed multifunctional nanocomposites, fluorescent-electrical-magnetic trifunctional coaxial nanoribbons with tunable fluorescent color, including white-light emission, have been successfully fabricated *via* coaxial electrospinning technology. Each stripe of coaxial nanoribbon is composed of a Fe_3_O_4_/PMMA core and a [Eu(BA)_3_phen+Dy(BA)_3_phen]/PANI/PMMA (PMMA = polymethyl methacrylate, BA = benzoic acid, phen = phenanthroline, polyaniline = PANI) shell. X-ray diffractometry (XRD), field emission scanning electron microscopy (FE-SEM), biological microscopy (BM), vibrating sample magnetometry (VSM), energy dispersive spectrometry (EDS), Hall effect measurement system and photoluminescence (PL) spectroscopy were employed to characterize the coaxial nanoribbons. Emitting color of the coaxial nanoribbons can be tuned by adjusting the contents of Dy(BA)_3_phen, Eu(BA)_3_phen, PANI and Fe_3_O_4_ in a wide color range of blue-white-orange under the excitation of 273-nm single-wavelength ultraviolet light. The coaxial nanoribbons simultaneously possess excellent luminescent performance, electrical conduction and magnetism compared with the counterpart composite nanoribbons. Furthermore, the electrical and magnetic performances of the coaxial nanoribbons also can be tunable by adding different quantities of PANI and Fe_3_O_4_ nanoparticles, respectively. The obtained coaxial nanoribbons have promising applications in many areas, such as electromagnetic interference shielding, microwave absorption, molecular electronics, biomedicine, future nanomechanics and display fields.

One dimensional (1D) multifunctional nanocomposites that possess desirable properties in a single entity have attracted broad interest in recent years^1^,^2^,^3^,^4^,^5^. They have bi- or trifunction such as electrical, optical, magnetic and chemical properties, leading to the wide range of technological applications in various fields. Among these nanocomposites, the magnetic-fluorescent bifunctional nanocomposites combined magnetic with fluorescent functionalities have particularly attracted great attention due to the unique properties associated with promising applications, such as targeted drug delivery^6^, magnetic resonance imaging (MRI)^7^, optical imaging^8^, etc. Electromagnetically functionalized micro/nanostructures of conducting polymers are of special interest due to their potential applications in areas such as electromagnetic interference shielding^9^, microwave absorption^10^, nonlinear optics^11^ and biomedicine^12^. Previously, polyaniline (PANI) nanotubes containing Fe_3_O_4_ nanoparticles (NPs) have been fabricated by an *in situ* chemical oxidation polymerization in the presence of Fe_3_O_4_ NPs^13^. Recently, Zhang, *et al.* developed a very simple chemical one-step method to prepare PANI/α-Fe_2_O_3_ nanofibers^14^,^15^. Luminescent-electrical materials are expected to possess many potential applications in areas such as color display, electromagnetic shielding, molecular electronics and biomedicine. M. Ghoswami, *et al.*^2^ synthesized polyaniline-cadmium sulfide nanocomposite using chemical oxidative method. This composite can be used in different optoelectronic purposes and it is a promising material with prospect of application in polymer light emitting diodes (PLED). Lun, *et al.*^16^ fabricated novel [Tb(BA)_3_phen+Eu(BA)_3_phen]/PANI/PVP composite nanofibers with tunable color-electricity bifunctionality *via* facile one-pot electrospinning technology. The prepared composite nanofibers have potential applications in many fields such as color display, molecular electronics and biomedicine.

It is therefore of considerable interest to develop the luminescent-electrical-magnetic trifunctional nanomaterials which have potential applications in molecular electronics, biomedicine, microwave absorption, electromagnetic shielding, etc^17^,^18^. At present, some preparations of trifunctional photoluminescence-electricity-magnetism 1D nanomaterials have been reported. Yu, *et al.*^19^ illustrated a route for fabricating Fe_3_O_4_@Au/PANI multifunctional nanocomposites with a core-shell structure, where the Fe_3_O_4_@Au nanoparticles are well dispersed in the PANI matrix. The obtained Fe_3_O_4_@Au/PANI nanocomposites exhibit integrated optical, electrical and magnetic properties. Lun, *et al.*^20^ prepared the tuned electricity, magnetism and fluorescent color trifunctional composite microbelts *via* one-pot electrospinning technique. The composite microbelts suffered heavy losses in fluorescent intensity, because Fe_3_O_4_ NPs were directly mixed with the RE luminescent compounds. From that study, the multifunctional composite nanomaterials suffered heavy losses in fluorescent intensity when Fe_3_O_4_ NPs were directly blended with the luminescent compounds. So rare earth complex must be effectively isolated from Fe_3_O_4_ NPs to ultimately reduce the impact of Fe_3_O_4_ NPs on the fluorescent property if the strong luminescence is achieved.

Among various luminescent materials, rare earth complexes doped inorganic/organic hybrids have good thermal and mechanical stabilities and processing property. Furthermore, they have excellent luminescent property on account of the antenna effect of ligands and the f-f electron transition of RE^3+^ ions. Nowadays, tunable multicolor, especially white-light emissions, can be successfully achieved *via* varying the doping ions and doping concentration. For example, Sun, *et al.*^21^ prepared Gd_3_Ga_5_O_12_: Yb^3+^/Tm^3+^/Ho^3+^ nanocrystals *via* a citric acid complex procedure. By means of adjusting the doping concentrations of Yb^3+^/Tm^3+^/Ho^3+^, the red-green-blue up-conversion luminescence was obviously changed. Yi, *et al.*^22^ synthesized NaCeF_4_: Ln^3+^ (Eu^3+^, Dy^3+^, Tb^3+^) nanorods with tunable multicolor output and bright white emissions *via* a typical hydrothermal method using oleic acid as the capping agent. Guan, *et al.*^23^ successfully synthesized multicolor emitting Tb^3+^ and/or Sm^3+^ doped NaGdF_4_ luminescent nanomaterials *via* an SDS-assisted one-step hydrothermal method. The as-prepared Tb^3+^ or Sm^3+^ doped samples showed strong green and yellow emission. Lun, *et al.*^16^ reported tunable fluorescent color-electricity bifunctional composite nanofibers prepared by electrospinning, and the emitting color of the composite nanofibers can be tuned in a wide color range of red-yellow-green by adjusting the mass ratio of terbium complexes and europium complexes, or by changing PANI content. More importantly, Guan, *et al.*^24^ fabricated a series of tunable luminescence NaGdF_4_: Dy^3+^, Eu^3+^ nanophosphors for the first time by a one-step hydrothermal method, and the products achieved tunable multicolor and warm white-light emission under the excitation of the 273-nm ultraviolet light.

Nanoribbon is a kind of nanomaterial of special morphology^25^,^26^. An increasing number of scientists have shown strong interest to research it owing to its anisotropy, large width-thickness ratio, unique optical, electrical and magnetic properties.

In this paper, we report assembling tunable fluorescent color-electricity-magnetism trifunctionality into [Fe_3_O_4_/PMMA]@{[Dy(BA)_3_phen+Eu(BA)_3_phen]/PANI/PMMA} coaxial nanoribbons. Of the coaxial nanoribbon, the core is composed of template PMMA containing Fe_3_O_4_ NPs, and the shell consists of PMMA containing RE (Dy, Eu) complexes and PANI. This new nanostructure can successfully help to realize the effective separation of Fe_3_O_4_ NPs from RE complexes, and it is expected that this new nanostructure will lead to trifunctional flexible nanoribbons with high luminescence intensity. Furthermore, tunable multicolor output and bright white-light emissions can be achieved through co-doping of Dy complex and Eu complex. To the best of our knowledge, these new structured flexible coaxial nanoribbons prepared by means of one-step electrospinning have not been reported in any literature. More importantly, the proposed design idea and construction technique are of universality for preparing other multifunctional one-dimensional nanoribbons. The structure, fluorescence, electricity and magnetism of the coaxial nanoribbons were systematically studied, and some new results were obtained.

## Materials and Methods

### Chemicals

Methylmethacrylate (MMA), benzoylperoxide (BPO), Eu_2_O_3_ (99.99%), Dy_2_O_3_(99.99%), benzoic acid (BA), 1,10-phenanthroline (phen), FeCl_3_·6H_2_O, FeSO_4_·7H_2_O, NH_4_NO_3_, polyethylene glycol (PEG, Mr≈20 000), ammonia, anhydrous ethanol, CHCl_3_ and dimethylformamide (DMF) were bought from Tianjin Tiantai Fine Chemical Co., Ltd. Anhydrous ethanol, aniline (ANI), (IS)-(+)-Camphor-10 sulfonic acid (CSA) and oleic acid (OA) were purchased from Sinopharm Chemical Reagent Co., Ltd. Ammonium persulfate (APS) was bought from Guangdong Xilong Chemical Co., Ltd. Nitric acid (HNO_3_, AR) was purchased from Beijing Chemical Works. All the reagents were of analytical grade and directly used as received without further purification. Deionized water was made in our own laboratory.

### Synthesis of Eu(BA)_3_phen and Dy(BA)_3_phen complexes

Eu(BA)_3_phen powders were synthesized according to the traditional method as described in the reference^27^. 1.7600 g of Eu_2_O_3_ was dissolved in an amount of concentrated nitric acid and then crystallized *via* evaporation of excess nitric acid and water by heating, and Eu(NO_3_)_3_·6H_2_O was acquired. Eu(NO_3_)_3_ ethanol solution was prepared by adding amount of anhydrous ethanol into the above Eu(NO_3_)_3_·6H_2_O. 3.6640 g of BA and 1.8020 g of phen were dissolved in ethanol. The Eu(NO_3_)_3_ ethanol solution was then added dropwise into the mixture solution of BA and phen with magnetic agitation at 60 °C for 3 h. The precipitates were collected by filtration and washed for three times using ethanol, and then dried in an electric oven at 60 °C for 12 h. The synthetic method of Dy(BA)_3_phen complex was similar to the above method, except that the using dosages of Dy_2_O_3_, BA and phen were 1.8650 g, 3.6600 g and 1.8000 g, respectively.

### Preparation of PMMA

PMMA used in this study was prepared by oxidative polymerization of MMA^28^. MMA (100 mL) and BPO (0.1000 g) were mixed in a 250 mL three-necked flask with a backflow device and stirred vigorously at 90–95 °C. When the viscosity of the solution reached a certain value just like that of glycerol, the heating was stopped and it was left to naturally cool down to room temperature. The obtained gelatinous solution was then loaded into test tubes, and the influx height was 5–7 cm. After that, the tubes were put in an electric vacuum oven for 48 h at 50 °C, and the gelatinous solution was then solidified. Finally, the temperature in the oven was raised to 110 °C for 2 h to terminate the reaction. The weight-average molecular weight and the degree of polymerization (DP) value of as-prepared PMMA are 9.7 × 10^4^ and 9.7 × 10^2^, respectively.

### Preparation of oleic acid modified Fe_3_O_4_ NPs

Fe_3_O_4_ NPs were obtained *via* a facile coprecipitation synthetic method^29^, and PEG was used as the protective agent to prevent the particles from aggregating. One typical synthetic procedure was as follows: 5.4060 g of FeCl_3_·6H_2_O, 2.7800 g of FeSO_4_·7H_2_O, 4.0400 g of NH_4_NO_3_ and 1.9000 g of PEG were added into 100 mL of deionized water to form a uniform solution under vigorous stirring at 50 °C. To prevent the oxidation of Fe^2+^, the reactive mixture was kept under argon atmosphere. After the mixture had been bubbled with argon for 30 min, 0.1 mol·L^−1^ of NH_3_·H_2_O was dropwise added into the mixture until the pH value was above 11. Then the system was continuously bubbled with argon for 20 min at 50 °C, and black precipitates were formed. The precipitates were collected from the solution by magnetic separation, washed for three times with deionized water, and then dried in an electric vacuum oven at 60 °C for 12 h. The as-prepared Fe_3_O_4_ NPs were then coated with oleic acid (OA) as below: 2.0000 g of the as-prepared Fe_3_O_4_ NPs were ultrasonically dispersed in 100 mL of deionized water for 20 min. The suspension was heated to 80 °C under argon atmosphere with vigorous mechanical stirring for 30 min and then 1 mL of OA was dropwise added. Reaction was stopped after heating and stirring the mixture for 40 min. The precipitates were collected from the solution by magnetic separation, washed with ethyl alcohol for three times, and then dried in an electric vacuum oven for 6 h at 60 °C.

### Preparations of spinning solutions for fabricating coaxial nanoribbons

Two different kinds of spinning solutions were prepared to fabricate coaxial nanoribbons. The spinning solution for the shell (denoted as spinning solution I) of coaxial nanoribbons was composed of Dy(BA)_3_phen, Eu(BA)_3_phen, PANI, PMMA, DMF and CHCl_3_, and detailed preparation process for spinning solution I was as following: certain amount of ANI and CSA, and 0.5 g of PMMA were dissolved in the mixed solution of 0.3 g of DMF and 6 g of CHCl_3_ with magnetic stirring for 48 h at room temperature (defined as solution A). Meanwhile, APS was used as an oxidant and dispersed into a mixed solution of 0.6 g of DMF and 3 g of CHCl_3_ with magnetic stirring for at least 2 h at room temperature (defined as solution B). Then solution A and B were both cooled down to 0 °C in an ice-bath. Subsequently, solution B was added dropwise into solution A under magnetic stirring. The final mixture was allowed to react at 0 °C for 24 h to produce PANI by the polymerization of aniline^30^,^31^. Then certain amounts of Eu(BA)_3_phen and Dy(BA)_3_phen complexes were added into the mixture under magnetic stirring for another 12 h at room temperature, thus spinning solution I for the shell was prepared.

Compared to Eu(BA)_3_phen, Dy(BA)_3_phen has weaker luminescence intensity. Therefore, in this study, we need firstly find the optimum concentration of Dy(BA)_3_phen in the shell to guarantee the luminescence intensity of Dy(BA)_3_phen to reach maximum, and then different amounts of Eu(BA)_3_phen are introduced into the shell to realize tunable color. In order to find the optimum concentration of Dy(BA)_3_phen, a series of Dy(BA)_3_phen/PANI/PMMA composite nanoribbons were fabricated. For performing this study, the mass percentage of ANI to PMMA was settled as 30%, the mass percentages of Dy(BA)_3_phen to PMMA were varied from 120% to 240%. The corresponding samples were marked as a, b, c, d and e. The compositions and contents of these composite nanoribbons were listed in Table 1. Based on photoluminescence analysis discussed in section “Photoluminescence property”, the mass percentage of Dy(BA)_3_phen to PMMA settled as 180% was adopted to prepare [Fe_3_O_4_/PMMA]@{[Dy(BA)_3_phen+Eu(BA)_3_phen]/PANI/PMMA} coaxial nanoribbons. The dosages of materials used for preparing spinning solution I were shown in Table 2.

The other spinning solution for the core of coaxial nanoribbons consisted of certain amounts of OA modified Fe_3_O_4_ NPs, 0.5 g of PMMA, 9 g of CHCl_3_ and 0.9 g of DMF (denoted as spinning solution II). Fe_3_O_4_ NPs were dispersed in DMF and CHCl_3_ with the assist of ultrasonics for 15 min, and then PMMA was added into the above solution under mechanical stirring. In order to investigate the impact of Fe_3_O_4_ NPs on the properties of coaxial nanoribbons, various contents of Fe_3_O_4_ NPs were introduced into spinning solution II. The compositions and contents of the spinning solutions were summarized in Table 3. The obtained coaxial nanoribbons were denoted as S_ax_@S_by_ (x = 1–9; y = 1–3) according to the corresponding spinning solution I and II.

### Preparations of tunable multicolor and white-light emissions luminescent-electrical-magnetic trifunctional coaxial nanoribbons

**Figure 1:**
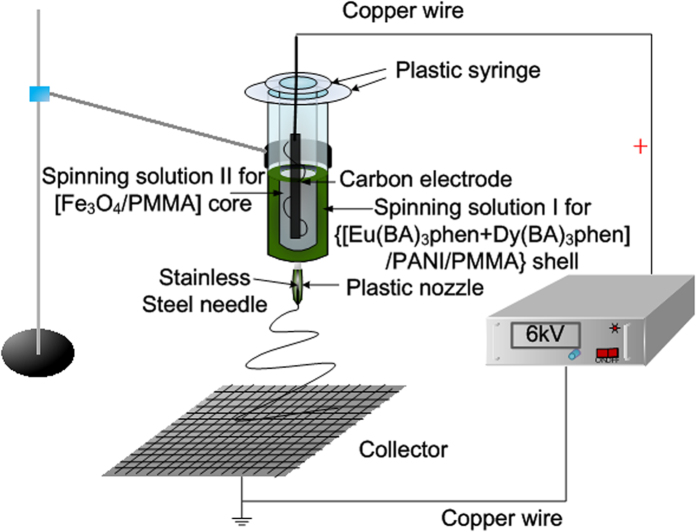
Schematic diagram of the equipment for electrospinning coaxial nanoribbons.

A homemade coaxial electrospinneret was used in this study. The equipment for the electrospinning process is presented in Fig. 1. Spinning solution I was loaded into the outer plastic syringe while spinning solution II was loaded into the inner plastic syringe. A piece of flat iron net was used as a collector and put about 10 cm away from the nozzle tip. The positive terminal of a direct current (DC) high voltage power supply was connected to the carbon electrode which was immersed into the spinning solution II, and the negative terminal was connected to the iron net. Positive DC voltage of 6 kV was applied between the nozzle and the collector to generate coaxial nanoribbons under the ambient temperature of 20–25 °C, and the relative humidity of 45–50%.

### Fabrication of Fe_3_O_4_/[Dy(BA)_3_phen+Eu(BA)_3_phen]/PANI/PMMA composite nanoribbons

In order to highlight advantages of coaxial nanostructure, Fe_3_O_4_/[Dy(BA)_3_phen+Eu(BA)_3_phen]/PANI/PMMA composite nanoribbons were also fabricated by mixing the spinning solution I-S_a3_ and spinning solution II-S_b1_ together at the volume ratio of 1:1. Preparation method is as follows: PANI was also obtained by the polymerization of 0.15 g of ANI, then 0.0025 g of Eu(BA)_3_phen, 0.9 g of Dy(BA)_3_phen and 0.5 g of Fe_3_O_4_ NPs were added into the as-prepared PANI solution under magnetic stirring for 12 h at room temperature. The spinning voltage, distance between the collector and the spinneret and other processing parameters were the same as they were in the fabrication of the [Fe_3_O_4_/PMMA]@{[Dy(BA)_3_phen+Eu(BA)_3_phen]/PANI/PMMA} coaxial nanoribbons. A traditional monoaxial electrospinneret was used to prepare the composite nanoribbons.

### Characterization

The as-prepared Fe_3_O_4_ NPs and [Fe_3_O_4_/PMMA]@{[Dy(BA)_3_phen+Eu(BA)_3_phen]/PANI/PMMA} coaxial nanoribbons were examined by X-ray powder diffractometer (XRD) performed on a Bruker, D8 FOCUS diffractometer with Cu Kα radiation (λ = 0.15406 nm) and Ni filter, the operation current and voltage were maintained at 20 mA and 40 kV, and scanning speed, step length and diffraction range were settled as 10° min^−1^, 0.1° and 20–70°, respectively. The morphology and size of Fe_3_O_4_ NPs were observed by a transmission electron microscope (TEM, JEOL, JEM-2010, Tokyo, Japan).The morphology and size of the coaxial nanoribbons were observed by a field emission scanning electron microscope (FESEM, XL-30) equipped with an energy-dispersive X-ray spectrometer (EDS). The internal structure of the coaxial nanoribbons was observed by a biological microscope (CVM500E). The measurements of photoluminescence (PL) spectra and the luminescence decay curves were performed by a HITACHI F-7000 fluorescence spectrophotometer using a 150 W Xe lamp as the excitation source, and scanning speed was fixed at 1200 nm·min^−1^. The excitation and emission slits were both set to 5.0 nm. Then, the magnetic performance of Fe_3_O_4_ NPs, coaxial nanoribbons and composite nanoribbons was measured by a vibrating sample magnetometer (VSM, MPMS SQUID XL). The conductive property was detected by Hall effect measurement system (ECOPIA HMS-3000). The ultraviolet-visible spectra of samples were determined by a UV-1240 ultraviolet-visible spectrophotometer. All the measures were performed at room temperature.

## Results and Discussion

### Crystallization behavior

**Figure 2:**
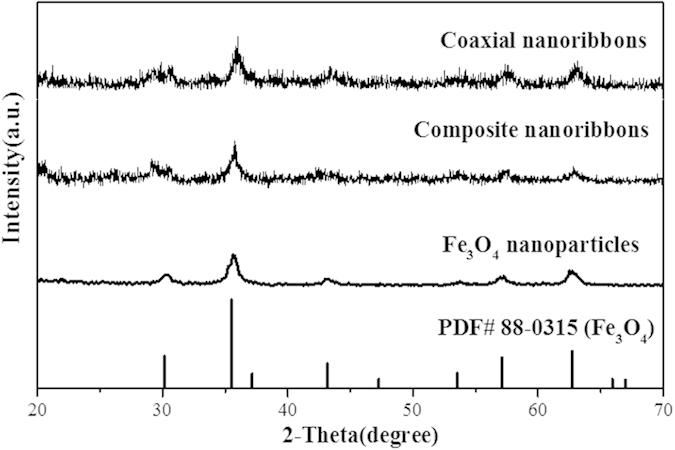
XRD patterns of Fe_3_O_4_ nanoparticles, [Fe_3_O_4_/PMMA]@{[Dy(BA)_3_phen+Eu(BA)_3_phen]/PANI/PMMA} coaxial nanoribbons (S_a3_@S_b2_) and composite nanoribbons with PDF standard card of Fe_3_O_4_.

XRD patterns of the as-obtained Fe_3_O_4_ NPs, [Fe_3_O_4_/PMMA]@{[Dy(BA)_3_phen+Eu(BA)_3_phen]/PANI/PMMA} coaxial nanoribbons (S_a3_@S_b2_) and composite nanoribbons are shown in Fig. 2. The diffraction peaks of the as-prepared Fe_3_O_4_ NPs can be well indexed to the cubic phase of Fe_3_O_4_ (PDF 88–0315). Obviously, only characteristic peaks of Fe_3_O_4_ existed in the sample, and no peak from the impurities such as Fe_2_O_3_ and FeO(OH) could be detected, indicating the formation of pure Fe_3_O_4_ phase. XRD analysis results of the coaxial nanoribbons and composite nanoribbons demonstrate that they contain Fe_3_O_4_ NPs, but the intensity is relatively lower due to the existence of other amorphous substances.

### Morphology and structure

**Figure 3:**
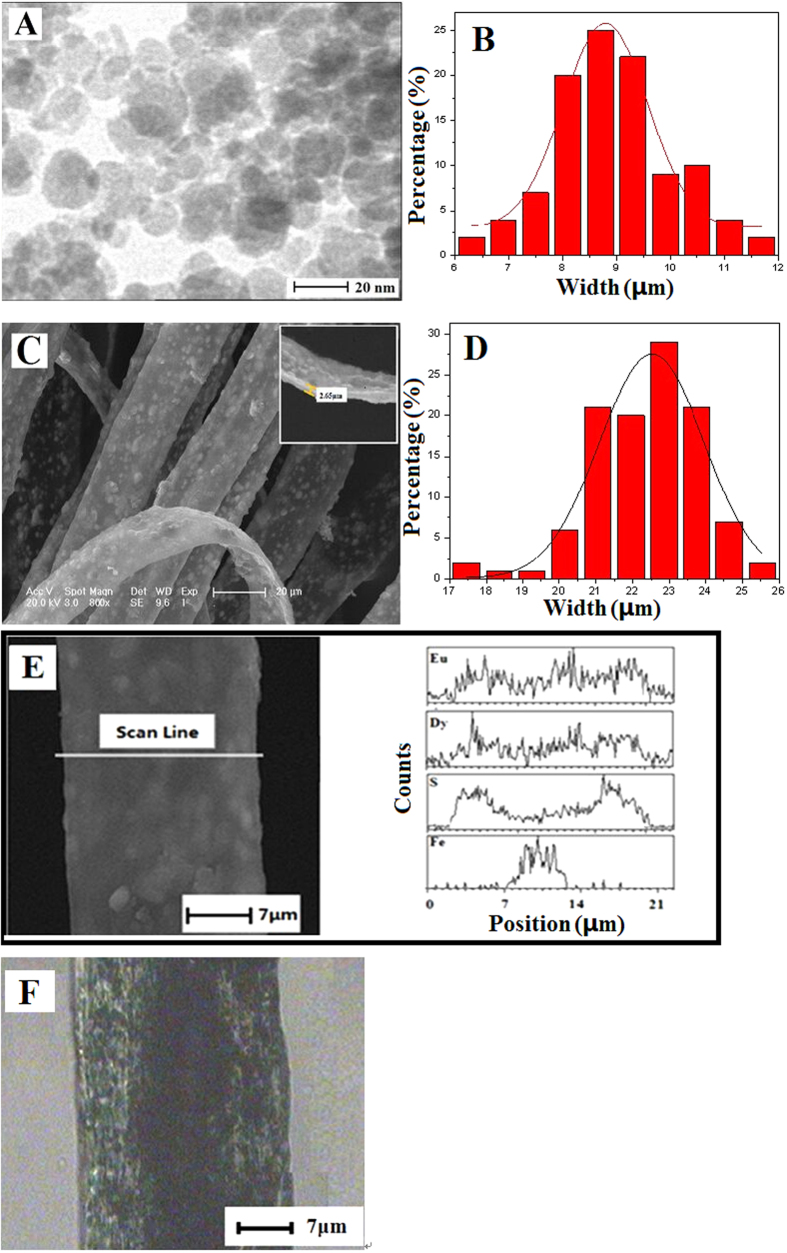
TEM image (**A**) and histogram of particle size of Fe_3_O_4_ NPs (**B**), SEM image (**C**), histogram of width (**D**), EDS line scan analysis (**E**) and BM image (**F**) of coaxial nanoribbons (S_a3_@S_b2_).

The morphology of the as-prepared Fe_3_O_4_ NPs was observed by means of TEM, as presented in Fig. 3A. The size distribution of the spherical Fe_3_O_4_ NPs is almost uniform, and the particle size of the Fe_3_O_4_ NPs is 8.79 ± 0.085 nm (Fig. 3B). The morphology and structure of [Fe_3_O_4_/PMMA]@{[Dy(BA)_3_phen+Eu(BA)_3_phen]/PANI/PMMA} coaxial nanoribbons (S_a3_@S_b2_) were characterized by a combination of SEM, BM and EDS line scan analysis. SEM image of the nanoribbons is shown in Fig. 3C. It is found that the as-prepared nanoribbons are relatively uniform, the width of the coaxial nanoribbons is 22.52 ± 0.153 μm (Fig. 3D) and the thickness is about 2.65 μm.

To further confirm the coaxial structure of the [Fe_3_O_4_/PMMA]@{[Dy(BA)_3_phen+Eu(BA)_3_phen]/PANI/PMMA} coaxial nanoribbons, EDS line-scan analysis was performed, in which Dy, Eu, S and Fe elements represent Dy(BA)_3_phen, Eu(BA)_3_phen, CSA doped PANI and Fe_3_O_4_, respectively, as shown in Fig. 3E. Elemental Fe only existed in the middle domain of the [Fe_3_O_4_/PMMA]@{[Dy(BA)_3_phen+Eu(BA)_3_phen]/PANI/PMMA} coaxial nanoribbons. The amounts of elemental Dy, Eu and S in the middle domain of the nanoribbons are lower than that in both sides of the nanoribbons because Dy(BA)_3_phen, Eu(BA)_3_phen, CSA doped PANI only exist in the top and bottom surfaces of the middle domain of the nanoribbons. It is further found that only elemental S without elemental Fe is dispersed in both sides of the coaxial nanoribbons. These results are consistent with the core-shell structure of coaxial nanoribbons.

Depending on the transmission light of the BM, the inner structure of the coaxial nanoribbons can also be observed. As revealed in Fig. 3F, a clear coaxial structure can be seen in the [Fe_3_O_4_/PMMA]@{[Dy(BA)_3_phen+Eu(BA)_3_phen]/PANI/PMMA} coaxial nanoribbons. The core of the coaxial nanoribbon is about 7 μm in width and it contains large quantities of dark-colored Fe_3_O_4_ NPs, and the shell of the coaxial nanoribbon is blackish green due to the existence of PANI.

From the SEM and BM observations and EDS line-scan analysis, we can safely draw a conclusion that [Fe_3_O_4_/PMMA]@{[Dy(BA)_3_phen+Eu(BA)_3_phen]/PANI/PMMA} coaxial nanoribbons have been successfully prepared.

### Photoluminescence property

**Figure 4:**
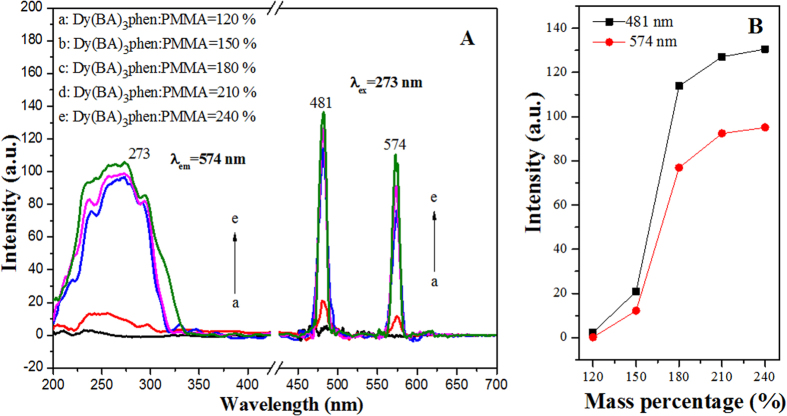
Excitation spectra (left) and emission spectra (right) of Dy(BA)_3_phen/PANI/PMMA composite nanoribbons containing different mass percentages of Dy(BA)_3_phen complex (A) and relationship between Dy(BA)_3_phen contents and intensities of luminescent peaks at 481 nm and 574 nm for Dy (BA)_3_phen/PANI/PMMA composite nanoribbons.

In order to find appropriate content of Dy(BA)_3_phen, a series of Dy(BA)_3_phen/PANI/PMMA composite nanoribbons were fabricated by electrospinning using different spinning solutions indicated in Table 1. The excitation and emission spectra of Dy(BA)_3_phen/PANI/PMMA composite nanoribbons are provided in Fig. 4. From the excitation spectra (Fig. 4A, left), a broad excitation band extending from 200 to 350 nm is observed from each sample when monitoring wavelength is 574 nm. The strongest peak at 273 nm assigned to the π→π* electron transition of the ligands could also be identified. Characteristic emission peaks of the Dy(BA)_3_phen are observed under the excitation of 273-nm ultraviolet light, which are ascribed to the energy levels transitions of ^4^F_9/2_→^6^H_15/2_ (481 nm) and ^4^F_9/2_→^6^H_13/2_ (574 nm). One can see that the photoluminescence intensity of Dy(BA)_3_phen/PANI/PMMA composite nanoribbons is increased with adding more Dy(BA)_3_phen. In order to further discuss the variation trend, the intensities of predominant emission peaks at 481 nm and 574 nm versus different mass percentages of Dy(BA)_3_phen to PMMA are plotted in Fig. 4B. Obviously, the fluorescence intensity only slightly increases with introducing more Dy(BA)_3_phen than the mass percentage of 180%. Therefore, the mass percentage of Dy(BA)_3_phen to PMMA settled as 180% was adopted to prepare [Fe_3_O_4_/PMMA]@{[Dy(BA)_3_phen+Eu(BA)_3_phen]/PANI/PMMA} coaxial nanoribbons.

**Figure 5:**
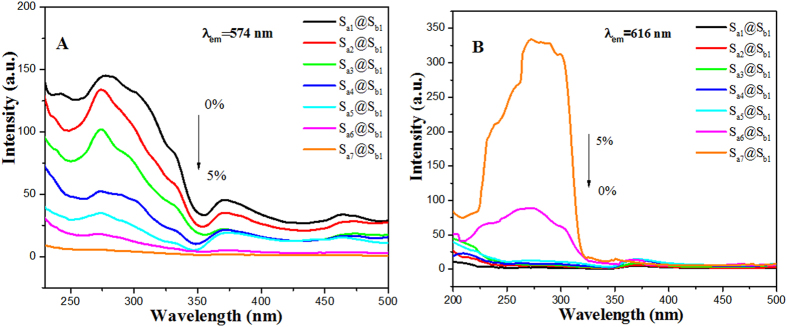
Excitation spectra of samples S_ax_@S_b1_ (x = 1–7) monitored at 574 nm (**A**) and 616 nm (**B**).

To study the color-tunable property of coaxial nanoribbons, the mass percentages of Eu(BA)_3_phen to PMMA were varied from 0 to 5% (samples S_ax_@S_b1_, x = 1–7), while the mass percentage of PANI to PMMA was settled as 30% and the mass ratio of Fe_3_O_4_ to PMMA was fixed as 1:1. Figure 5A shows excitation spectra of coaxial nanoribbons monitored at 574 nm, where 574 nm is the characteristic emission wavelength of Dy^3+^. Figure 5B demonstrates the excitation spectra of the samples monitored at 616 nm, where 616 nm is the characteristic emission wavelength of Eu^3+^. The strongest peak at 273 nm assigned to the π→π* electron transition of the ligands could be also identified, and the excitation intensity is increased with introducing more Eu(BA)_3_phen. The blue shift of excitation peak of Dy^3+^ is probably due to the strong absorption of Eu(BA)_3_phen around 273 nm, resulting in the decrease of light absorption of Eu(BA)_3_phen around 273 nm.

**Figure 6:**
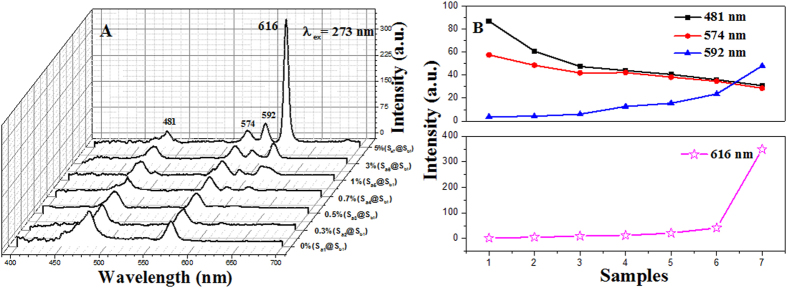
Emission spectra of samples S_ax_@S_b1_ (x = 1–7) when the mass percentage of PANI is fixed as 30% and the mass ratio of Fe_3_O_4_ to PMMA is fixed as 1:1 (**A**), dependence of the emission intensity on the various samples (**B**).

Figure 6A displays the emission spectra of sample S_ax_@S_b1_ (x = 1–7). Upon excitation with 273-nm ultraviolet light, coaxial nanoribbons exhibit several main emission bands, whose positions locate at 481 nm, 574 nm, 592 nm and 616 nm. It is found that the emissions at 481 and 574 nm are due to the energy levels transitions of ^4^F_9/2_→^6^H_J/2(J = 15,13)_ of Dy^3+^ and the peaks at 592 and 616 nm are corresponding to the energy levels transitions of ^5^D_0_→^7^F_1_ and ^5^D_0_→^7^F_2_ of Eu^3+^, respectively. It is interesting and reasonable to suggest that the emission intensity of Eu^3+^ is increased, whereas that of the Dy^3+^ is simultaneously found to decrease monotonically with the increase of Eu^3+^ ions concentration. In order to clearly depict the variation trend, the intensities of the characteristic emission peaks of each sample versus different samples were plotted in the Fig. 6B. The variation of the PL intensity of the Eu^3+^ and Dy^3+^ can be attributed to the energy distribution. Since the energy that the matrix absorbs and the content of Dy(BA)_3_phen are constant, more energy is assigned to Eu^3+^ with the increase of Eu(BA)_3_phen content, thus leading to stronger fluorescence peaks at 592 and 616 nm. Meanwhile, on the contrary, the energy assigned to Dy^3+^ is reduced and the fluorescence peaks at 481 and 574 nm are relevantly weakened.

**Figure 7:**
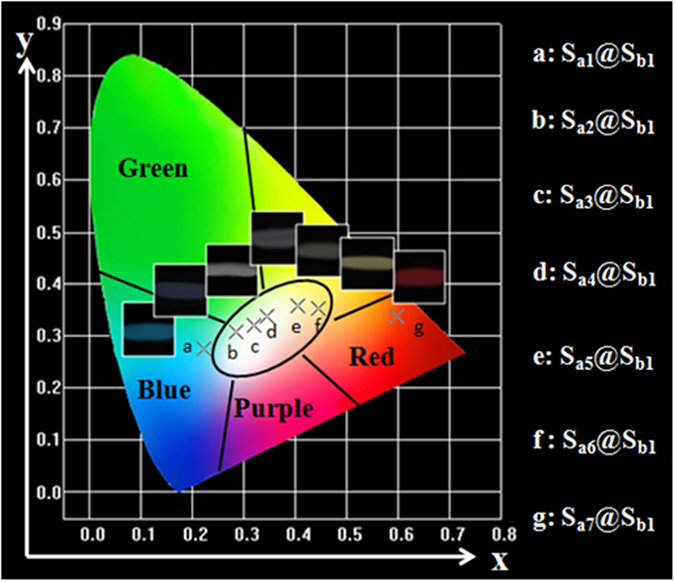
CIE chromaticity coordinates diagram of samples S_ax_@S_b1_ (x = 1–7) and the corresponding luminescence photographs under the excitation of 273-nm ultraviolet light.

Generally, color can be represented by the Commission Internationale de L’Eclairage (CIE) chromaticity coordinates. The CIE chromaticity coordinates for the samples and their corresponding photographs upon excitation at 273-nm ultraviolet light are provided in Table 4 and Fig. 7. It is found that the emitting color of coaxial nanoribbons (sample S_ax_@S_b1_ x = 1–7) could be tuned by adjusting the mass ratio of Eu(BA)_3_phen complexes in a wide color range of blue-white-orange. Among all the coaxial nanoribbons, the coaxial nanoribbons with 0.7% Eu^3+^ (sample S_a4_@S_b1_) have coordinates of x = 0.344, y = 0.337 which are close to those of standard white light (x = 0.333, y = 0.333), indicating that the as-obtained coaxial nanoribbons can emit warm white-light color. It is gratify to see that the warm white emission can be selectively realized by the co-doping of Dy(BA)_3_phen and Eu(BA)_3_phen complexes into coaxial nanoribbons.

**Figure 8:**
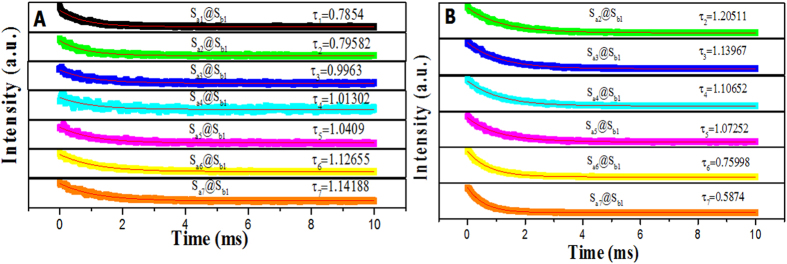
Fluorescence decay dynamics of the ^4^F_9/2_→^6^H_13/2_ transitions of Dy^3+^ (λ_em _= 574 nm) (**A**) and the ^5^D_0_→^7^F_2_ transitions of Eu^3+^ (λ_em _= 616 nm) (B) in samples S_ax_@S_b1_ (x = 1–7).

The fluorescence lifetime curves of Dy^3+^ emission at 574 nm and Eu^3+^ emission at 616 nm in samples S_ax_@S_b1_ (x = 1–7) under the excitation of 273-nm ultraviolet light are shown in Fig. 8 (A,B). From Fig. 8(A,B), one can see that all the luminescent decay lifetimes fit the single exponential rule by the following equation as the Fig. 8 (A,B) depicts.


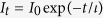


Where *I*_*t*_ and *I*_*0*_ are the luminescence intensities at times t and 0, respectively, t is the decay time and *τ* is the lifetime. Figure 8A shows that fluorescence decay lifetime of the ^4^F_9/2_→^6^H_13/2_ transitions (λ_em _= 574 nm) in coaxial nanoribbons is extended with increasing in Eu^3+^ concentration, while fluorescence decay lifetime of the ^5^D_0_→^7^F_2_ transitions (λ_em _= 616 nm) decreases, as shown in Fig. 8B. On one hand, the relative content of Dy(BA)_3_phen complex in the coaxial nanoribbons is reduced with introducing more Eu(BA)_3_phen. Thus the distance among Dy^3+^ in Dy(BA)_3_phen molecular clusters and/or nanoparticles in the coaxial nanoribbons is increased, resulting in that the energy transfer among Dy^3+^ to Dy^3+^ is reduced and the fluorescence lifetime of Dy^3+^ is prolonged. On the other hand, more aggregates of Eu(BA)_3_phen are formed in the polymer matrix with introducing more Eu(BA)_3_phen. The exciton migration between the Eu(BA)_3_phen molecules shortens the fluorescence lifetime of Eu^3+^ Ref. 32.

**Figure 9:**
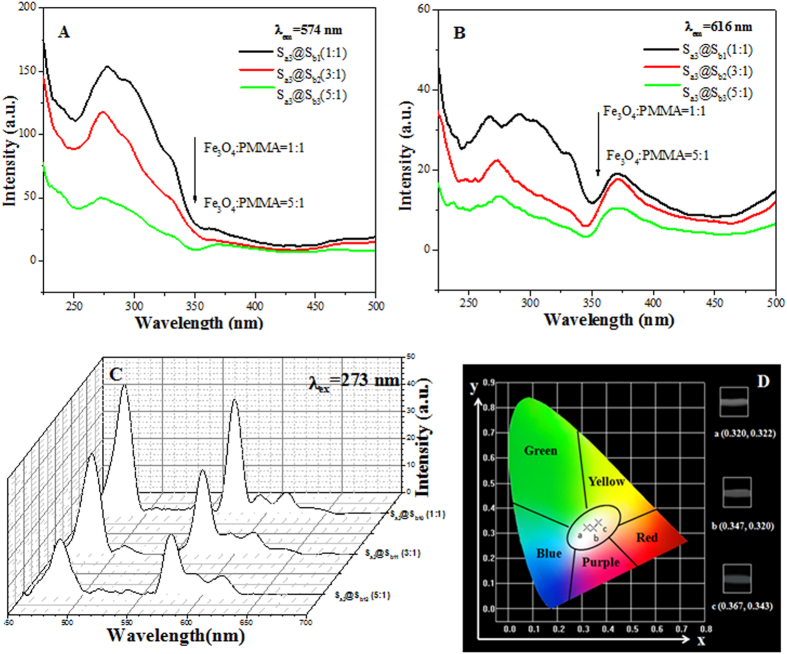
Excitation spectra (**A,B**), emission spectra (**C**) and CIE chromaticity coordinate diagram (**D**) of [Fe_3_O_4_/PMMA]@{[Dy(BA)_3_phen+Eu(BA)_3_phen]/PANI/PMMA} coaxial nanoribbons containing different mass percentages of Fe_3_O_4_.

**Figure 10:**
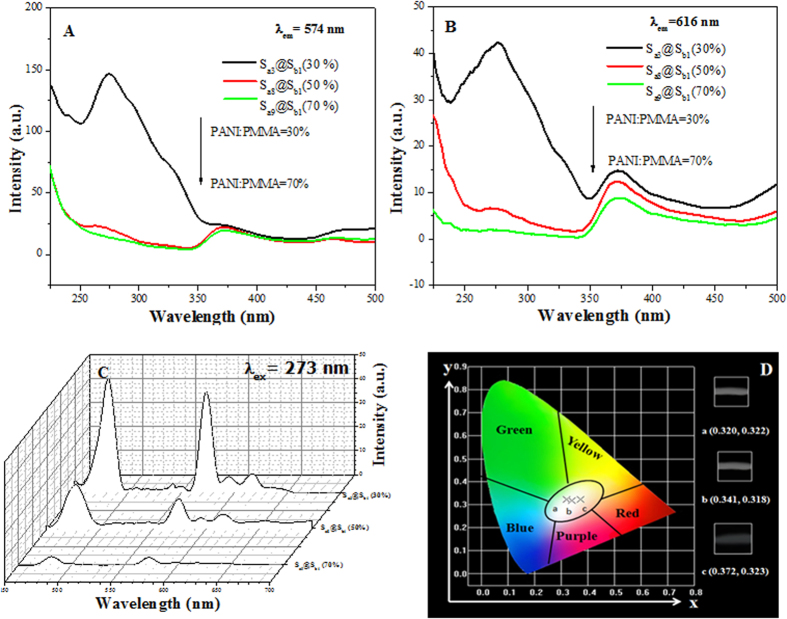
Excitation spectra (**A,B**), emission spectra (**C**) and CIE chromaticity coordinate diagram (**D**) of [Fe_3_O_4_/PMMA]@{[Dy(BA)_3_phen+Eu(BA)_3_phen]/PANI/PMMA} coaxial nanoribbons containing different mass percentages of PANI.

Meanwhile, the coaxial nanoribbons containing different amounts of PANI and Fe_3_O_4_ NPs were fabricated to research the effect of adding different contents of Fe_3_O_4_ NPs (samples S_a3_@S_b1_, S_a3_@S_b2_, S_a3_@S_b3_, as illustrated in Fig. 9) and PANI (samples S_a3_@S_b1_, S_a8_@S_b1_, S_a9_@S_b1_, as shown in Fig. 10) on the fluorescent properties of the coaxial nanoribbons. As shown in Fig. 9 (A–C), the excitation and emission intensity of coaxial nanoribbons are decreased with the increase of Fe_3_O_4_ NPs content. Figure 9D is the CIE chromaticity coordinate diagram of coaxial nanoribbons with different Fe_3_O_4_ NPs contents under the excitation of 273-nm ultraviolet light. It demonstrates that the emitting color of the coaxial nanoribbons shifts with introducing more Fe_3_O_4_ NPs. Similarly, when the amount of PANI is increased, the excitation and emission intensity of coaxial nanoribbons are decreased as illustrated in Fig. 10 (A–C). The CIE chromaticity coordinates for the samples and their corresponding photographs upon excitation at 273-nm ultraviolet light are provided in the Fig. 10D. It is found that the emitting color of coaxial nanoribbons could be shifted by adjusting the mass ratio of PANI.

**Figure 11:**
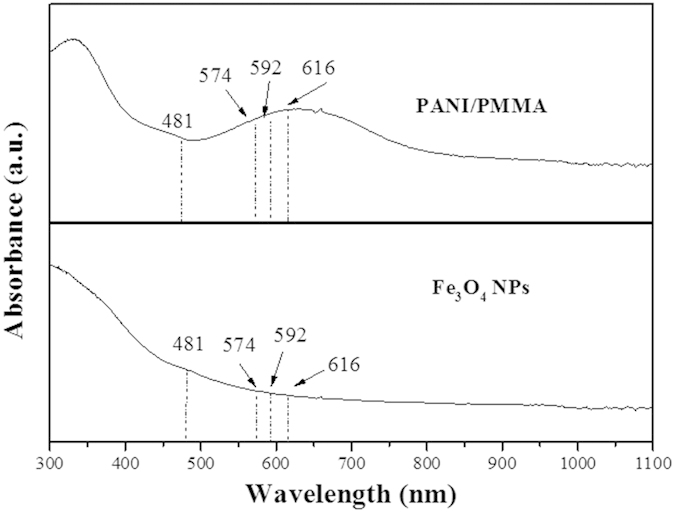
Ultraviolet-visible absorption spectra of PANI/PMMA and Fe_3_O_4_ NPs.

The above results can be explained as the light absorption of Fe_3_O_4_ NPs and PANI. From the absorption spectra of Fe_3_O_4_ NPs and PANI illustrated in Fig. 11, it is seen that PANI doped PMMA strongly absorb the light in the regions of ultraviolet light (<400 nm) and 400–800 nm, and Fe_3_O_4_ NPs can absorb visible light (400–760 nm) and much more easily absorb the ultraviolet light (<400 nm). Thus, the exciting light and emitting light are absorbed by the PANI and Fe_3_O_4_ NPs, resulting in the decrease in the intensity of excitation and emission peaks. Moreover, the light absorbance becomes stronger with more PANI and Fe_3_O_4_ NPs introduced into coaxial nanoribbons. On the other hand, because PANI has different absorbance to different wavelengths of light, as well as Fe_3_O_4_ NPs, as seen in Fig. 11, different wavelengths of light emitted from coaxial nanoribbons are unequally absorbed by PANI and Fe_3_O_4_, leading to the fact that the emitting colors are shifted.

**Figure 12:**
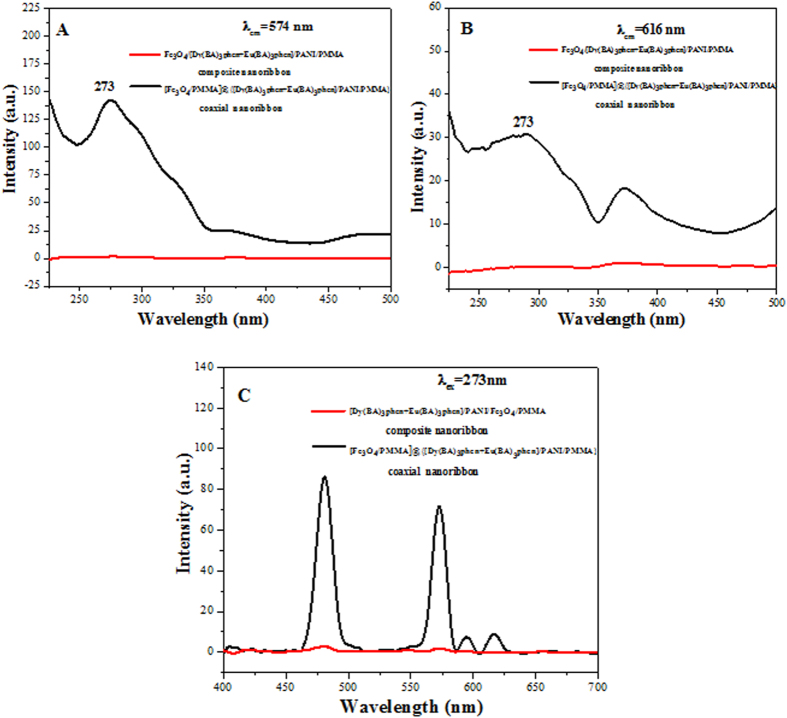
Excitation spectra (**A,B**) and emission spectra (**C**) of [Fe_3_O_4_/PMMA]@{[Dy(BA)_3_phen+Eu(BA)_3_phen]/PANI/PMMA} coaxial nanoribbons (S_a3_@S_b1_) and Fe_3_O_4_/[Dy(BA)_3_phen+Eu(BA)_3_phen]/PANI/PMMA composite nanoribbons.

**Figure 13:**
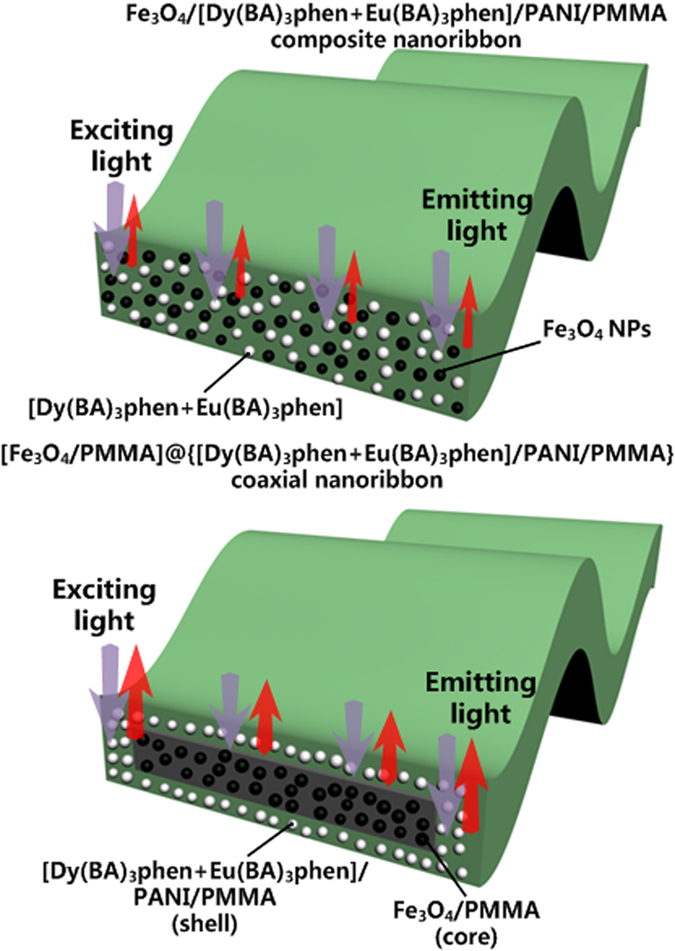
Schematic diagrams of the situation of the exciting light and emitting light in the Fe_3_O_4_/[Dy(BA)_3_phen+Eu(BA)_3_phen]/PANI/PMMA composite nanoribbons and [Fe_3_O_4_/PMMA]@{[Dy(BA)_3_phen+Eu(BA)_3_phen]/PANI/PMMA} coaxial nanoribbons.

The fluorescent properties of coaxial nanoribbons are further investigated by comparing with that of the Fe_3_O_4_/[Dy(BA)_3_phen+Eu(BA)_3_phen]/PANI/PMMA composite nanoribbons. As manifested in Fig. 12, it is found that the emission and excition intensity of the Fe_3_O_4_/[Dy(BA)_3_phen+Eu(BA)_3_phen]/PANI/PMMA composite nanoribbons is much weaker than that of the coaxial nanoribbons, and this weak fluorescent emission intensity makes the Fe_3_O_4_/[Dy(BA)_3_phen+Eu(BA)_3_phen]/PANI/PMMA composite nanoribbons impractical in fluorescent performance. This heavy loss in fluorescent emission intensity is from the strong light absorption of the dark-colored Fe_3_O_4_ NPs when Fe_3_O_4_ NPs are directly blended with Dy(BA)_3_phen and Eu(BA)_3_phen. As illustrated in Fig. 13, since the Fe_3_O_4_ NPs are distributed over the whole parts of the Fe_3_O_4_/[Dy(BA)_3_phen+Eu(BA)_3_phen]/PANI/PMMA composite nanoribbons, the exciting light has to pass through many Fe_3_O_4_ NPs to reach the Dy(BA)_3_phen and Eu(BA)_3_phen complexes. In this process, a large part of the exciting light has been absorbed by the Fe_3_O_4_ NPs, so the exciting light is much weakened before it reaches the Dy(BA)_3_phen and Eu(BA)_3_phen complexes. Similarly, the light emitted by the Dy(BA)_3_phen and Eu(BA)_3_phen complexes also has to pass through the Fe_3_O_4_ NPs and is absorbed by them, which results in the emitting light being severely weakened. For the coaxial nanoribbons, Fe_3_O_4_ NPs are separated from Dy(BA)_3_phen and Eu(BA)_3_phen complexes in their own domains of the coaxial nanoribbons, so that the exciting light and emitting light in the {[Dy(BA)_3_phen+Eu(BA)_3_phen]/PANI/PMMA} domain will almost be unaffected by Fe_3_O_4_ NPs. The overall result is that the coaxial nanoribbons possess much higher fluorescent performance than the Fe_3_O_4_/[Dy(BA)_3_phen+Eu(BA)_3_phen]/PANI/PMMA composite nanoribbons. Thus, a strong fluorescent emission intensity of the coaxial nanoribbons is achieved by isolating Dy(BA)_3_phen and Eu(BA)_3_phen from Fe_3_O_4_ NPs.

### Electrical conductivity analysis

The conductivity of coaxial nanoribbons can be tuned by adjusting the mass percentage of PANI to PMMA. It is found from Table 5 that the electrical conductivities of products are 9.42 × 10^−4^ S·cm^−1^, 4.26 × 10^−3^ S·cm^−1^ and 1.47 × 10^−2^ S·cm^−1^ when percentages of PANI is 30 wt%, 50 wt % and 70 wt %, respectively. Obviously, the more PANI introduced into the coaxial nanoribbons, the higher electrical conductivity of the electrospun coaxial nanoribbons. As PANI is consecutive in the shell of coaxial nanoribbons and probably forms the conducting network more easily, which renders more efficient charge transport.

The conductivity of coaxial nanoribbon (sample S_a3_@S_b1_) is nearly slightly bigger than that of Fe_3_O_4_/[Dy(BA)_3_phen+Eu(BA)_3_phen]/PANI/PMMA composite nanoribbons under the same components and contents of the two kinds of nanostructures. The reason is probably that the insulative materials such as Fe_3_O_4_ NPs and RE(BA)_3_phen (RE = Dy, Eu) are dispersed in the Fe_3_O_4_/[Dy(BA)_3_phen+Eu(BA)_3_phen]/PANI/PMMA composite nanoribbons, which hinders the formation of continuous conductive network.

### Magnetic property

**Figure 14:**
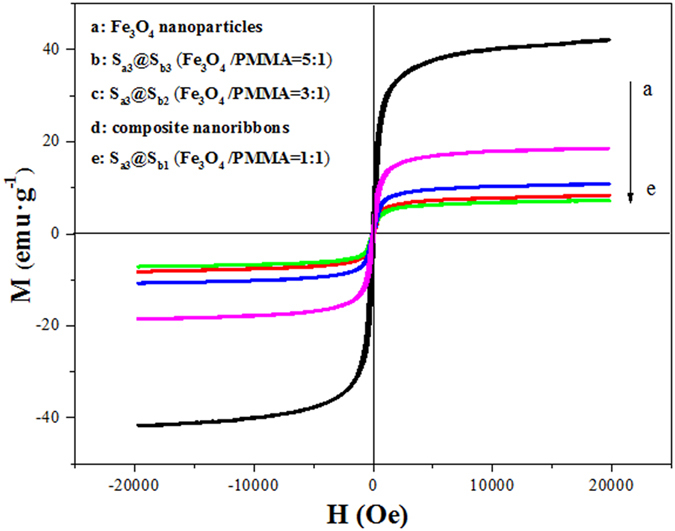
Hysteresis loops of Fe_3_O_4_ NPs, Fe_3_O_4_/[Dy(BA)_3_phen+Eu(BA)_3_phen]/PANI/PMMA composite nanoribbons and [Fe_3_O_4_/PMMA]@{[Dy(BA)_3_phen+Eu(BA)_3_phen]/PANI/PMMA} coaxial nanoribbons.

The typical hysteresis loops for Fe_3_O_4_ NPs, Fe_3_O_4_/[Dy(BA)_3_phen+Eu(BA)_3_phen]/PANI/PMMA composite nanoribbons and [Fe_3_O_4_/PMMA]@{[Dy(BA)_3_phen+Eu(BA)_3_phen]/PANI/PMMA} coaxial nanoribbons containing various mass ratios of Fe_3_O_4_ NPs are shown in Fig. 14, and the saturation magnetizations of them are listed in Table 6. It is found that the saturation magnetization of the [Fe_3_O_4_/PMMA]@{[Dy(BA)_3_phen+Eu(BA)_3_phen]/PANI/PMMA} coaxial nanoribbons is increased with the increase of the amount of Fe_3_O_4_ NPs in the core. Besides, the saturation magnetization of the Fe_3_O_4_/[Dy(BA)_3_phen+Eu(BA)_3_phen]/PANI/PMMA composite nanoribbons is 8.38 emu·g^−1^ which is close to that of the coaxial nanoribbons (7.23 emu·g^−1^, S_a3_@S_b1_). By combining the analyses of magnetism, electrical conductivity and fluorescence, it is found that the coaxial nanoribbons have close magnetic property compared with composite nanoribbons, while the fluorescent intensity and electrical conductivity of the coaxial nanoribbons are much higher than those of the composite nanoribbons, and those further demonstrate that the coaxial nanoribbons possess better luminescent-electrical-magnetic performance than the counterpart composite nanoribbons. Based on the above experimental results, we can safely conclude that the shortcomings of the existing luminescence-electricity-magnetism 1D nanomaterials described in the introduction have been greatly overcome.

## Conclusions

[Fe_3_O_4_/PMMA]@{[Dy(BA)_3_phen+Eu(BA)_3_phen]/PANI/PMMA} coaxial nanoribbons with tunable fluorescent color, magnetism and electricity trifunctionality were successfully synthesized by electrospinning technology using a specially designed coaxial spinneret. The core of the coaxial nanoribbons is composed of Fe_3_O_4_ NPs and PMMA, and the shell consists of Dy(BA)_3_phen, Eu(BA)_3_phen, PANI and PMMA. Under the excitation of 273-nm single-wavelength ultraviolet light, the emitting color of the coaxial nanoribbons can be tuned in a wide color range of blue-white-orange by adjusting the mass ratio of Dy(BA)_3_phen and Eu(BA)_3_phen complexes. More significantly, warm white luminescent color can be achieved. It is also found that PANI and Fe_3_O_4_ NPs affect luminescent color as well. The luminescent intensity, electrical conductivity and magnetic property of the coaxial nanoribbons can be tunable by adjusting the contents of RE complexes, PANI and Fe_3_O_4_ NPs, respectively. It is very exciting to see that the coaxial nanoribbons simultaneously possess excellent luminescent performance, electrical conduction and magnetic properties. The new high-performance luminescent-electrical-magnetic trifunctional coaxial nanoribbons have potential applications in molecular electronics, microwave absorption, color display device and future nanodevice.

## Additional Information

**How to cite this article**: Shao, H. *et al.* Electrospun Flexible Coaxial Nanoribbons Endowed With Tuned and Simultaneous Fluorescent Color-Electricity-Magnetism Trifunctionality. *Sci. Rep.* **5**, 14052; doi: 10.1038/srep14052 (2015).

## Figures and Tables

**Table 1:** Compositions of Dy(BA)_3_phen/PANI/PMMA composite nanoribbons.

Samples	Compositions	
	Dy(BA)_3_phen/g	PMMA/g
a	0.60	0.5
b	0.75	0.5
c	0.90	0.5
d	1.05	0.5
e	1.20	0.5

**Table 2:** Compositions of the spinning solution I.

Spinning solutions	Composition				
	Eu(BA)_3_phen/g	Dy(BA)_3_phen/g	ANI/g	CSA/g	APS/g
S_a1_	0	0.9	0.15	0.2809	0.3676
S_a2_	0.0015	0.9	0.15	0.2809	0.3676
S_a3_	0.0025	0.9	0.15	0.2809	0.3676
S_a4_	0.0035	0.9	0.15	0.2809	0.3676
S_a5_	0.0150	0.9	0.15	0.2809	0.3676
S_a6_	0.0250	0.9	0.15	0.2809	0.3676
S_a7_	0.0350	0.9	0.15	0.2809	0.3676
S_a8_	0.0025	0.9	0.25	0.4682	0.6126
S_a9_	0.0025	0.9	0.35	0.6554	0.8578

**Table 3:** Compositions of the spinning solution II.

Spinning solutions	Composition			
	Fe_3_O_4_/g	PMMA/g	CHCl_3_/g	DMF/g
S_b1_	0.5	0.5	9	0.9
S_b2_	1.5	0.5	9	0.9
S_b3_	2.5	0.5	9	0.9

**Table 4:** Comparison among the CIE chromaticity coordinates (x, y) for the coaxial nanoribbons excited by 273-nm ultraviolet light.

Samples	Eu(BA)_3_phen concentration (n%)	CIE coordinates (x, y)
S_a1_@S_b1_	0	(0.223, 0.274)
S_a2_@S_b1_	0.3	(0.285, 0.308)
S_a3_@S_b1_	0.5	(0.320, 0.322)
S_a4_@S_b1_	0.7	(0.344, 0.337)
S_a5_@S_b1_	1.0	(0.405, 0.357)
S_a6_@S_b1_	3.0	(0.445, 0.353)
S_a7_@S_b1_	5.0	(0.598, 0.338)

**Table 5:** Electrical conductivity and resistivity of the samples doped with various amount of PANI.

Samples	Conductivity (S·cm^−1^)	Resistivity (Ω·cm)
S_a3_@S_b1_	9.42 × 10^−4^	1.062 × 10^3^
S_a8_@S_b1_	4.26 × 10^−3^	2.35 × 10^2^
S_a9_@S_b1_	1.47 × 10^−2^	6.8 × 10^1^
Composite nanoribbons	6.03 × 10^−4^	1.658 × 10^3^

**Table 6:** Saturation magnetization of Fe_3_O_4_ NPs, Fe_3_O_4_/[Dy(BA)_3_phen+Eu(BA)_3_phen]/PANI/PMMA composite nanoribbons and [Fe_3_O_4_/PMMA]@{[Dy(BA)_3_phen+Eu(BA)_3_phen]/PANI/PMMA} coaxial nanoribbons.

Samples	Saturation magnetization (Ms)/(emu·g^−1^)
Fe_3_O_4_ nanoparticles	42.17
Composite nanoribbons	8.38
S_a3_@S_b1_ (Fe_3_O_4_ : PMMA = 1:1)	7.23
S_a3_@S_b2_ (Fe_3_O_4_ : PMMA = 3:1)	10.8
S_a3_@S_b3_ (Fe_3_O_4_ : PMMA = 5:1)	18.58
